# Comprehensive RNA-Seq Analysis on the Regulation of Tomato Ripening by Exogenous Auxin

**DOI:** 10.1371/journal.pone.0156453

**Published:** 2016-05-26

**Authors:** Jiayin Li, Xiaoya Tao, Li Li, Linchun Mao, Zisheng Luo, Zia Ullah Khan, Tiejin Ying

**Affiliations:** 1 College of Biosystems Engineering and Food Science, Fuli Institute of Food Science, Zhejiang Key Laboratory for Agro-Food Processing, Zhejiang R & D Center for Food Technology and Equipment, Zhejiang University, Hangzhou, People’s Republic of China; 2 Department of Agriculture, Abdul Wali Khan University, Mardan, Khyber-Pakhtunkhwa, Pakistan; Iwate University, JAPAN

## Abstract

Auxin has been shown to modulate the fruit ripening process. However, the molecular mechanisms underlying auxin regulation of fruit ripening are still not clear. Illumina RNA sequencing was performed on mature green cherry tomato fruit 1 and 7 days after auxin treatment, with untreated fruit as a control. The results showed that exogenous auxin maintained system 1 ethylene synthesis and delayed the onset of system 2 ethylene synthesis and the ripening process. At the molecular level, genes associated with stress resistance were significantly up-regulated, but genes related to carotenoid metabolism, cell degradation and energy metabolism were strongly down-regulated by exogenous auxin. Furthermore, genes encoding DNA demethylases were inhibited by auxin, whereas genes encoding cytosine-5 DNA methyltransferases were induced, which contributed to the maintenance of high methylation levels in the nucleus and thus inhibited the ripening process. Additionally, exogenous auxin altered the expression patterns of ethylene and auxin signaling-related genes that were induced or repressed in the normal ripening process, suggesting significant crosstalk between these two hormones during tomato ripening. The present work is the first comprehensive transcriptome analysis of auxin-treated tomato fruit during ripening. Our results provide comprehensive insights into the effects of auxin on the tomato ripening process and the mechanism of crosstalk between auxin and ethylene.

## Introduction

Fruit ripening is a complex and highly coordinated process, which includes rapid changes in color, texture and flavor. Ethylene and auxin are two important classes of phytohormones that have been reported to modulate fruit ripening [1–3]. Ethylene is the most important phytohormone in the climacteric fruit ripening process, and its function has been well documented [4–6]. Recent reports have indicated that the interaction between ethylene and auxin may be crucial for fruit ripening [7–9].

In some fruits, such as strawberry, grape and tomato, application of auxin in the pre-ripening stage delayed ripening [9–11]. However, in pear and apple, applying auxin to the whole tree instead of the fruits before the onset of ripening increased ethylene production and induced earlier softening, leading to the acceleration of ripening [12–14]. In tomato and grape berry, low levels of auxin were essential for triggering the onset of ripening, whereas elevated auxin coincided with the burst of ethylene during the ripening period [8, 15]. These results suggest that auxin not only acts as an inhibitor but also plays complex roles in modulating fruit ripening.

Auxin and ethylene have been shown to interact with each other to regulate many physiological processes [16, 17]. The transcriptional accumulation of many auxin response factors (*ARF*) and auxin/indole-3-acetic acid (*Aux/IAA*) genes in tomato is controlled by both ethylene and auxin in tomato seedlings [18–20]. Similarly, the expression of ethylene response factor (*ERF*) genes can also be regulated by auxin [21].

The genome sequencing of tomato (*Solanum lycopersicum*), a good model plant for investigating fruit development, has provided powerful insights into the molecular changes in fruit ripening [22]. However, the role of auxin in the fruit ripening process has not been fully elucidated, although the involvement of auxin response genes in the regulation of ripening has been demonstrated [23–25]. Moreover, the molecular mechanism and details of the crosstalk between auxin and ethylene are also ambiguous. To date, few comprehensive transcriptome studies have been conducted on auxin-treated tomato fruit during ripening. Thus, we employed Illumina RNA sequencing to analyze cherry tomato fruit 1 and 7 days after exogenous auxin application to elucidate the molecular regulatory mechanisms of auxin and its interplay with ethylene during the ripening period. The alteration in phytohormone signal transduction and ripening-related metabolic pathways were investigated, and the short-term and long-term effects of auxin on fruit ripening were determined. The results provide comprehensive insights into the regulatory mechanisms of exogenous auxin on tomato ripening.

## Materials and Methods

### Plant materials and treatments

Mature green cherry tomato fruit (*Solanum lycopersicum* cv. *Xin Taiyang*) with a uniform shape and size was collected from a standard greenhouse (20–25°C, 70%-85% RH) from Transfar Agribio Co., Ltd. in Xiaoshan County, Zhejiang Province, China. Fruits were sterilized by dipping them in 0.5% (v/v) sodium hypochlorite aqueous solution for 5 min, washing twice with sterile water and air-drying at room temperature. After removing pedicels, the fruits were randomly divided into two groups, then immersed into 0.45 mM 2, 4-dichlorophenoxyacetic acid or sterile water and infiltrated under a vacuum (35 kPa, 3 min). After treatment, the fruits were stored in the dark at 20 ± 2°C with 90 ± 5% relativity humidity (RH) for 25 days. Samples were taken at 0, 1, 4, 7, 10, 13, 15, 18, 21, 25 days after treatment (DAT). Pericarps of the sampled fruit were cut into pieces, frozen in liquid nitrogen and kept at -70°C for subsequent use.

### Fruit color and texture

Five fruits from each group were used to measure fruit color and texture. Fruit color was expressed using the l*, a*, b* color space coordinates and measured with a Chroma meter (Konica Minolta, CR-400, Japan) at four symmetrical locations around the equator, as previously described [26]. Each color value was obtained from five fruits. Hue angle (*H°*) was calculated as *H°* = arctangent (b*/a*) × (180/π) to evaluate the color change. The hue angles near 120° and 0° represent pale green and red, respectively. Fruit firmness was measured by pushing a probe (5 mm diameter) into the pericarp at a speed of 1 mm·s^-1^ to a depth of 12 mm using a Texture Analyzer (TA-XT2i, Stable Microsystems Texture Technologies Inc., UK). The assessment was performed at two opposite locations along the fruit equator after peeling off the epidermis. The maximum force was recorded to represent fruit firmness.

### Ethylene measurement

Ethylene was assayed as previously described [27]. Twenty fruits from the control or auxin groups were sealed in a 2000 mL jar and kept at 20°C in the dark for 2 h. Ethylene concentration was measured by injecting l mL headspace gas into a gas chromatograph (model SP 6800, Lunan Chemical Engineering Instrument Co., China) equipped with a GDX-502 column (JieDao TECH, China) and a flame ionization detector (Shimadzu GC-2014C, Shimadzu Corporation, Japan). Measurements were performed in triplicate.

### ACO (1-aminocyclopropane-1-carboxylate oxidase) activity assay

ACO activity was measured using the method of Mei Zhang *et al*. [28] with slight modifications. Pericarp samples (1 g) were homogenized with 3 mL pre-chilled extract solution [100 mM Tris-HCl, pH 7.5, 10% (w/v) glycerin, 5% (w/v) polyvinylpyrrolidone, 5 mM DDT, 30 mM sodium ascorbic acid, 0.1 mM FeSO_4_] and centrifuged at 12,000 × *g* (4°C) for 10 min. The supernatant (0.5 mL) was added to 1.5 mL of the reaction solution (10% glycerin, 30 mM sodium ascorbic acid, 2 mM 1-aminocyclopropane-1-carboxylic acid, 0.1 mM FeSO_4_) in a 10 mL tube, sealed with a rubber plug and incubated at 30°C for 60 min. Ethylene in the head space was measured by gas chromatography. ACO activity was expressed as nmol C_2_H_4_ h^-1^ g^-1^ FW.

### Library preparation and Illumina sequencing

Fruits from each group were sampled and denoted CK1d (samples from the control group at 1 DAT), CK7d (samples from the control group at 7 DAT), AX1d (samples from the auxin group at 1 DAT), or AX7d (samples from the auxin group at 7 DAT). Two biological replicates, each containing pooled pericarps from eight fruits, were prepared for the RNA-Seq assays. Library preparation and transcriptome sequencing were performed by Novogene Bioinformatics Technology Co., Ltd. (Beijing, China). Total RNA was isolated with TRIzol® LS Reagent as described by the manufacturer’s protocol. RNA concentration was measured using a Qubit® RNA Assay Kit with a Qubit® 2.0 Fluorometer (Life Technologies, CA, USA), and integrity was assessed using the RNA Nano 6000 Assay Kit with a Bioanalyzer 2100 system (Agilent Technologies, CA, USA).

A total of 3 μg of RNA per sample was used as the input material for the RNA sample preparations. Sequencing libraries were generated using a NEBNext® Ultra™ RNA Library Prep Kit for Illumina® (NEB, USA) following the manufacturer’s recommendations, and index codes were added to attribute sequences to each sample. The clustering of the index-coded samples was performed on a cBot Cluster Generation System using TruSeq PE Cluster Kit v3-cBot-HS (Illumina, USA) according to the manufacturer’s instructions. After cluster generation, the library preparations were sequenced on an Illumina HiSeq 2000 platform, and 100 bp paired-end reads were generated.

### Transcriptome analysis

Clean data (clean reads), which were obtained by removing low quality reads from the raw data, were mapped to the tomato genome assembly SL2.50 using TopHat v2.0.2 [29]. Reads per kilobase of exon model per million mapped reads (RPKM) were calculated using HTSeq v0.6.1 to estimate gene expression levels [30].

Differential expression analysis of two groups was performed using the DESeq R package. *P*-values were adjusted by *Q*-values using the Benjamini-Hochberg method [31] for controlling the false discovery rate. Genes with *Q*-values < 0.05 were defined as differentially expressed.

Gene Ontology (GO) enrichment analysis of differentially expressed genes was performed using the GOseq R package as described by Young, M. D., *et al*. [32], in which gene length bias was corrected. GO terms with corrected *P*-values less than 0.05 were considered significantly enriched by differentially expressed genes. KOBAS software was used to test the statistical enrichment of differentially expressed genes in the Kyoto Encyclopedia of Genes and Genomes (KEGG) pathways as previously described [33].

### Quantitative real-time PCR assay

Total RNA was extracted using RNAiso plus (TaKaRa, Japan) according to the manufacturer’s protocol. cDNA was obtained from 1 μg of total RNA using a PrimeScript RT kit (TaKaRa, Japan). Quantitative real-time PCR assay was performed using SYBR® Premix Ex Taq (TaKaRa, Japan) on an ABI StepOne Real-Time PCR System (Applied Biosystems, USA) as previously described [27]. The tomato β-actin gene was used as the reference gene to calculate relative expression levels based on the 2^-ΔΔCt^ method [34]. Primers are listed in S1 Table.

### Correlation network analysis and statistics

Correlation network analysis was performed as previously described [35]. The network was visualized with Cytoscape version 3.2.1 (www.cytoscape.org) [36]. For biological and biochemical data, significant differences between samples were determined with a Student's *t*-test (independent samples) using SPSS version 20.0 (IBM Corp, Armonk, USA).

## Results

### Effects of auxin treatment on postharvest fruit color, firmness and ethylene synthesis

**Fig 1:**
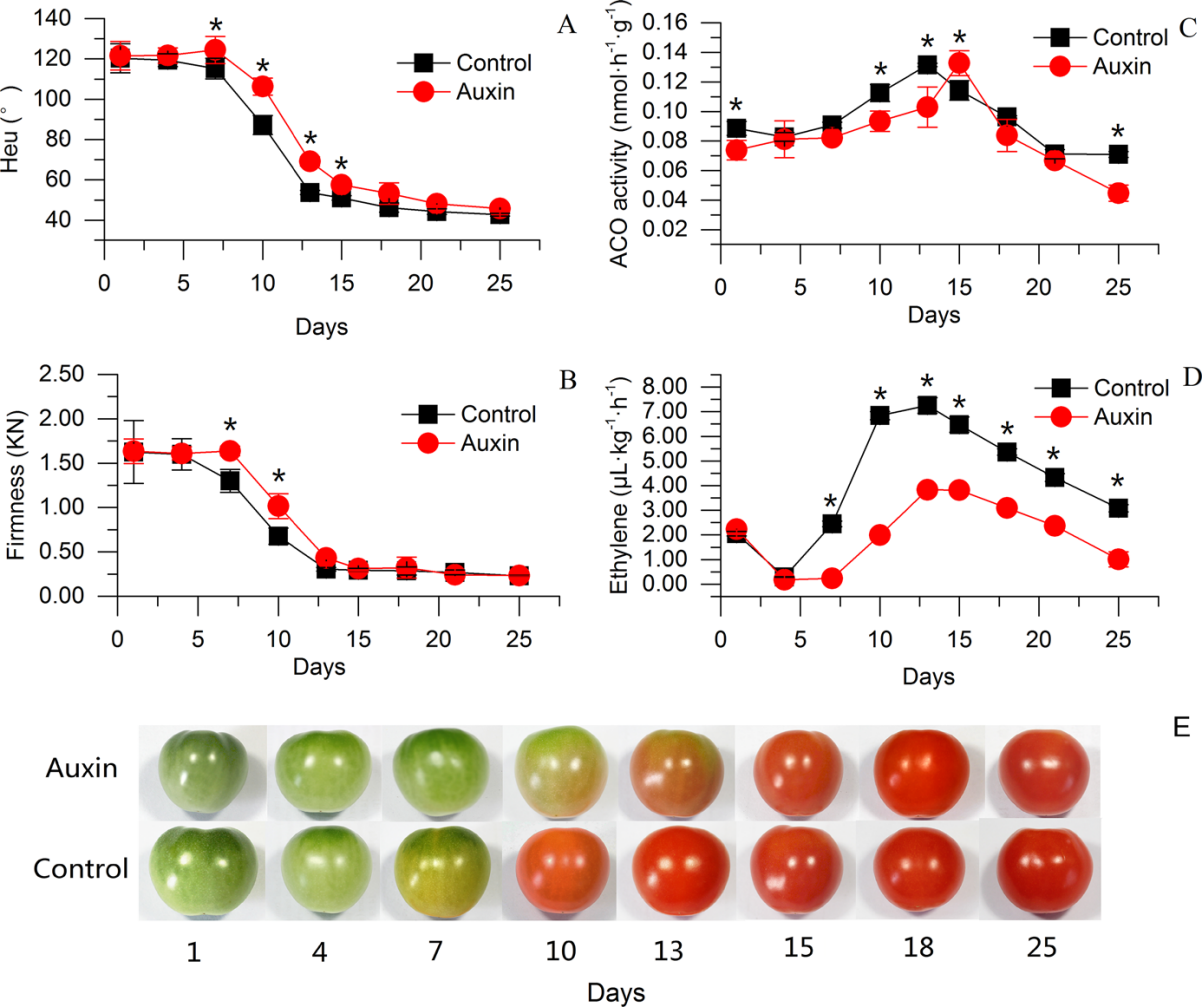
Physiological changes induced by exogenous auxin application in tomato fruit. The changes in (A) hue angle, (B) firmness, (C) ACO activity, (D) and ethylene and the pictures of tomato fruits after auxin treatment. Error bars indicate the standard error of three replicates. Asterisks (*) represent significant differences between the control and auxin treatments (Student's *t*-test, *P* < 0.05).

A significant inhibition in ripening was observed in auxin-treated fruit during storage (Fig 1E). The control fruit began to turn orange at 7 DAT coupled with a rapid softening, and the fruit turned red at 15 DAT. Changes in the fruit color and texture were delayed by auxin treatment. The auxin-treated fruit remained green and hard until 10 DAT (Fig 1A and 1B).

Ethylene production was lower in auxin-treated fruit than that in control fruit during storage (Fig 1D). Auxin treatment delayed the initiation of climacteric ethylene for approximately 3 days compared with the control. ACO activity was also inhibited by auxin during the first two weeks after treatment, although the maximum activity was approximately the same in the control and auxin-treated fruits (Fig 1C).

### Summary of transcriptome sequencing data

To elucidate the short-term and long-term effects of auxin treatment, fruits in both the auxin-treated and control groups were sampled at 1 day and 7 days after treatment for RNA-Seq analysis. Raw data generated by sequencing ranged from 30.3 to 37.6 million reads per sample, and more than 95% of them had a quality score ≥ Q20. After filtering, 29.9 to 37.0 million clean reads were obtained, approximately 91% of which could be mapped to the tomato reference genome. In addition, more than 90% of the clean reads were uniquely mapped reads, while the proportion of multiple mapped reads was less than 0.8% (Table 1).

**Table 1:** Throughput and the quality of RNA-Seq data.

Sample name	Raw reads	Clean reads	Q20 (%)	Mapped reads	Mapped percent	Uniquely mapped reads	Multiple mapped reads
**CK1d_1**	37652220	37030234	96.67	68154908	(92.03%)	67623746 (91.31%)	531162 (0.72%)
**CK1d_2**	30654627	30272262	96.35	55865924	(92.27%)	55467758 (91.61%)	398166 (0.66%)
**AX1d_1**	30329099	29900210	95.71	54636510	(91.36%)	54284505 (90.78%)	352005 (0.59%)
**AX1d_2**	30862471	30397218	95.94	55769863	(91.74%)	55392789 (91.11%)	377074 (0.62%)
**CK7d_1**	33350282	32878536	95.74	60241973	(91.61%)	59851967 (91.02%)	390006 (0.59%)
**CK7d_2**	31573280	31090705	95.66	56743198	(91.25%)	56333250 (90.60%)	409948 (0.66%)
**AX7d_1**	35514663	34990712	95.87	63657461	(90.96%)	63271623 (90.41%)	385838 (0.55%)
**AX7d_2**	33332070	32802692	95.78	60215602	(91.78%)	59839662 (91.21%)	375940 (0.57%)

Q20 and Q30 represent the percentage of the sequencing data with an error rate less than 1% and 0.1%, respectively.

**Fig 2:**
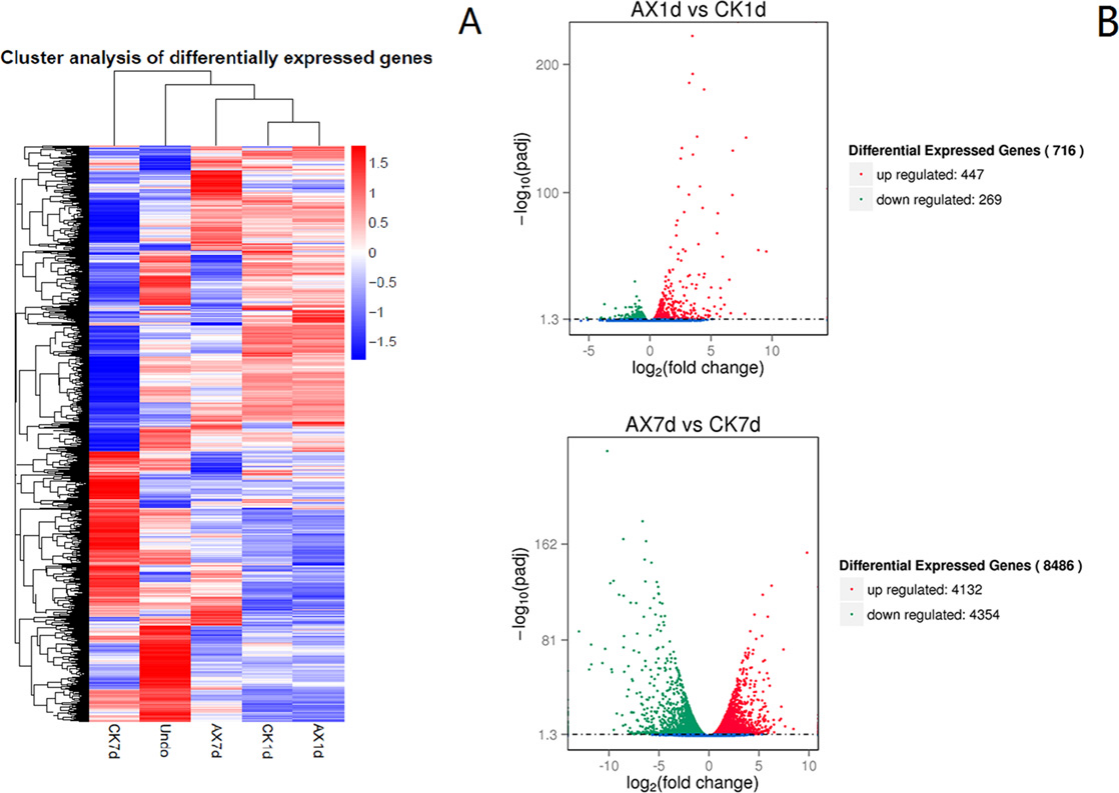
Differentially expressed genes in the samples. (A) Hierarchical clustering and heat map of differentially expressed genes based on the expression levels (RPKM). Genes in red and blue represent highly and lowly expressed genes, respectively. (B) The volcano plot shows the numbers of significantly differentially expressed genes in each comparison group.

More than 34,000 genes with different abundances were detected, and approximately 50% of them had a RPKM ≥1 (S2 Table). Hierarchical cluster analysis was used to estimate the variance in differentially expressed genes (DEGs), and we obtained 16,157 DEGs in the samples (Fig 2A). Compared with the control, 447 and 4132 genes were up-regulated by auxin at 1 and 7 DAT, while 269 and 4354 genes were repressed, respectively (Fig 2B, S3 and S4 Tables).

### GO enrichment and KEGG pathway analysis of DEGs

**Fig 3:**
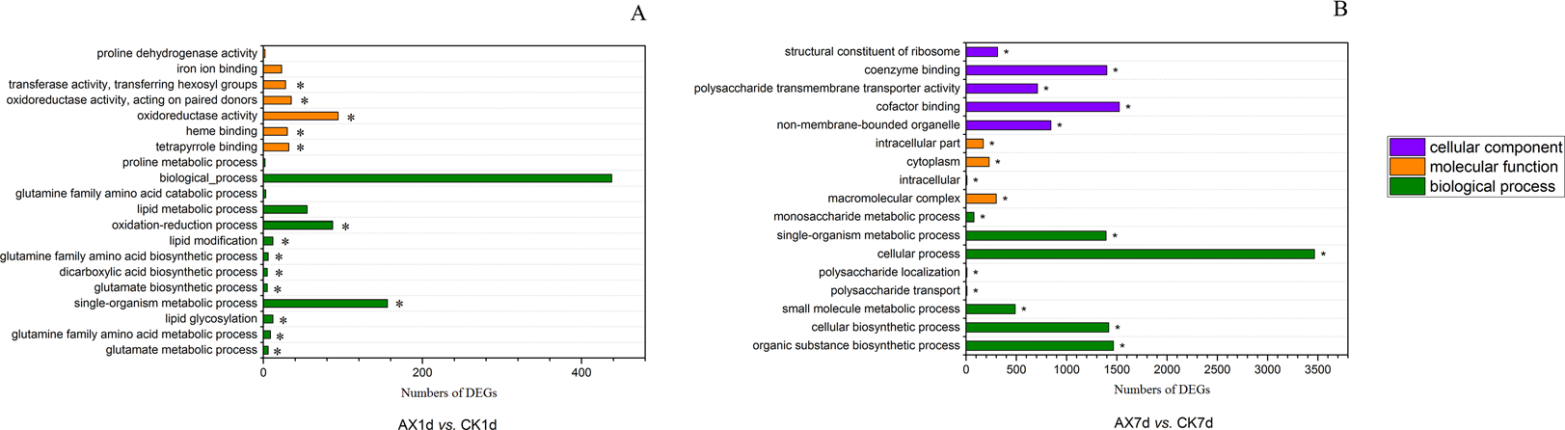
GO enrichment analysis of DEGs in all comparison groups. The top twenty most enriched GO terms in the comparison groups (A) AX1d *vs*. CK1d (B) and AX7d *vs*. CK7d. Asterisks (*) indicate significantly (*Q*-value < 0.05) enriched GO terms.

We performed GO enrichment analysis to investigate the distribution of DEGs in biological process (BP), cellular component (CC) and molecular function (MF). Fourteen GO terms (BP: 9 terms, MF: 5 terms) were significantly enriched in AX1d compared with CK1d, and glutamate metabolic process (GO: 0006536, 6 DEGs) and tetrapyrrole binding (GO: 0046906, 32 DEGs) were the most significantly enriched terms in BP and MF, respectively (Fig 3A). In addition, fifty-six GO terms (BP: 35 terms, MF: 6 terms, CC: 13 terms) were significantly enriched in AX7d compared with CK7d. The most significantly enriched terms in BP, MF and CC were cellular metabolic process (GO: 0044237, 2658 DEGs), cofactor binding (GO: 0048037, 301 DEGs) and macromolecular complex (GO: 0032991, 843 DEGs), respectively (Fig 3B). Detailed results of the GO enrichment analysis are shown in S5 and S6 Tables.

**Fig 4:**
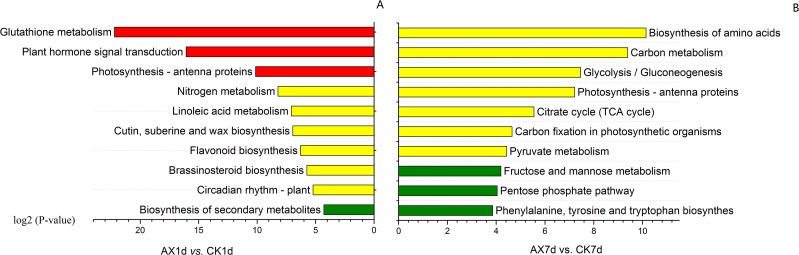
KEGG enrichment analysis of DEGs in all comparison groups. The top ten most enriched KEGG pathways in the comparison groups (A) AX1d *vs*. CK1d and (B) AX7d *vs*. CK7d. The bars in red, yellow and dark green represent the KEGG pathways with different enrichment levels (*Q*-value < 0.05, *P*-value < 0.05 but *Q*-value > 0.05, *P*-value > 0.05, respectively).

To investigate the major pathways of the DEGs, we aligned all DEGs to KEGG pathways (S7 and S8 Tables). In the AX1d *vs*. CK1d group, DEGs were enriched in 84 KEGG metabolic pathways and “Metabolic pathways” (75 DEGs), “Biosynthesis of secondary metabolites” (49 DEGs) and “Plant hormone signal transduction” (28 DEGs) were the top three pathways containing the greatest number of DEGs. Among the 84 pathways, nine had *P-*values > 0.05, and “Glutathione metabolism”, “Plant hormone signal transduction”, and “Photosynthesis-antenna proteins” were the most significantly enriched pathways (*Q*-value < 0.05) (Fig 4). In the AX7d *vs*. CK7d group, one hundred and twenty-three KEGG metabolic pathways were identified, and the top three pathways containing the greatest number of DEGs were “Metabolic pathways” (861 DEGs), “Biosynthesis of secondary metabolites” (471 DEGs) and “Ribosome” (158 DEGs). Seven KEGG pathways had *P-*values < 0.05, and “Biosynthesis of amino acids” (144 DEGs), “Carbon metabolism” (151 DEGs), “Glycolysis/Gluconeogenesis” (77 DEGs) were the major enriched pathways (Fig 4).

### Expression of genes involved in ethylene synthesis and signal transduction

**Fig 5:**
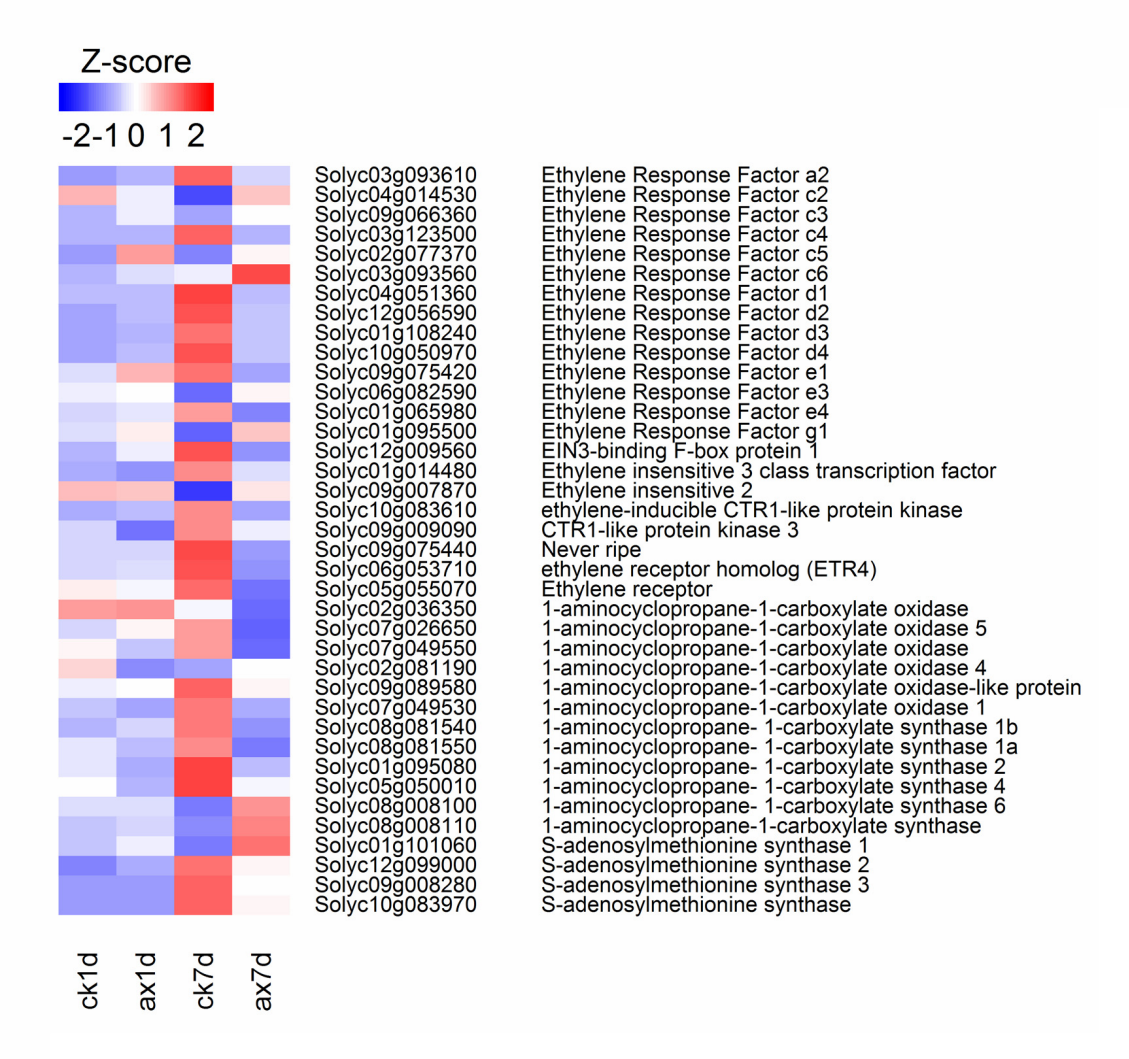
Heat map of the expression levels of the DEGs involved in ethylene synthesis and signal transduction. The RPKM values were normalized with log_2_ (RPKM+1) and converted to *Z*-scores to scale the expression levels of DEGs involved in ethylene synthesis and signal transduction. Red indicates high expression, while blue denotes low expression.

Thirty-nine genes related to ethylene synthesis and signal transduction were differentially expressed among the samples (Fig 5, Table 2). Compared with the control, the expression levels of SlACS4 (Solyc05g050010) and SlACO4 (Solyc02g081190) were reduced in auxin-treated samples at 1 DAT. However, at 7 DAT, one *SAM (*S-adenosylmethionine synthase) gene (Solyc01g101060) was induced by exogenous auxin, while the other three (Solyc10g083970, Solyc09g008280, Solyc12g099000) were down-regulated. ACS (1-aminocyclopropane-1-carboxylate synthase) and ACO (1-aminocyclopropane-1-carboxylate oxidase) are two important enzymes in ethylene biosynthesis. In this experiment, four *ACS* genes (Solyc05g050010, Solyc01g095080, Solyc08g081550, Solyc08g081540) and six *ACO* genes (Solyc07g049530, Solyc09g089580, Solyc02g081190, Solyc07g049550, Solyc07g026650, Solyc02g036350) were variously down-regulated in AX7d, but two *ACS* genes (Solyc08g008110, Solyc08g008100) were significantly up-regulated, although ethylene biosynthesis was repressed (Fig 1D).

**Table 2:** DEGs involved in ethylene synthesis and signal transduction.

		Fold change (log_2_ ratio)	
Gene ID	Annotation	AX1d *vs*.CK1d	AX1d *vs*.CK1d
Solyc10g083970	S-adenosylmethionine synthase	-	-1.76
Solyc09g008280	S-adenosylmethionine synthase 3	-	-2.74
Solyc12g099000	S-adenosylmethionine synthase 2	-	-1.24
Solyc01g101060	S-adenosylmethionine synthase 1	-	1.47
Solyc08g008110	1-aminocyclopropane-1-carboxylate synthase	-	3.86
Solyc08g008100	1-aminocyclopropane-1-carboxylate synthase 6	-	3.45
Solyc05g050010	1-aminocyclopropane-1-carboxylate synthase 4	-2.00	-4.48
Solyc01g095080	1-aminocyclopropane-1-carboxylate synthase 2	-	-5.01
Solyc08g081550	1-aminocyclopropane-1-carboxylate synthase 1a	-	-2.48
Solyc08g081540	1-aminocyclopropane-1-carboxylate synthase 1b	-	-2.34
Solyc07g049530	1-aminocyclopropane-1-carboxylate oxidase 1	-	-3.77
Solyc09g089580	1-aminocyclopropane-1-carboxylate oxidase-like protein	-	-2.25
Solyc02g081190	1-aminocyclopropane-1-carboxylate oxidase 4	-1.10	-
Solyc07g049550	1-aminocyclopropane-1-carboxylate oxidase	-	-5.96
Solyc07g026650	1-aminocyclopropane-1-carboxylate oxidase 5	-	-2.24
Solyc02g036350	1-aminocyclopropane-1-carboxylate oxidase	-	-0.87
Solyc05g055070	Ethylene receptor	-	-1.44
Solyc06g053710	Ethylene receptor homolog (ETR4)	-	-1.94
Solyc09g075440	Never ripe	-	-3.00
Solyc09g009090	CTR1-like protein kinase 3	-	-0.46
Solyc10g083610	Ethylene-inducible CTR1-like protein kinase	-	-1.20
Solyc09g007870	Ethylene insensitive 2	-	0.66
Solyc01g014480	Ethylene insensitive 3 class transcription factor	-	-0.58
Solyc07g008250	EIN3-binding F-box protein		-2.13
Solyc12g009560	EIN3-binding F-box protein 1	-	-3.07
Solyc01g095500	Ethylene response factor g1	-	3.03
Solyc01g065980	Ethylene response factor e4	-	-1.02
Solyc06g082590	Ethylene response factor e3	-	2.67
Solyc09g075420	Ethylene response factor e1	-	-2.06
Solyc10g050970	Ethylene response factor d4	-	-4.43
Solyc01g108240	Ethylene response factor d3	-	-3.92
Solyc12g056590	Ethylene response factor d2	-	-2.20
Solyc04g051360	Ethylene response factor d1	-	-9.52
Solyc03g093560	Ethylene response factor c6	-	1.40
Solyc02g077370	Ethylene response factor c5	1.93	1.28
Solyc03g123500	Ethylene response factor c4	-	-1.71
Solyc09g066360	Ethylene response factor c3	-	2.13
Solyc04g014530	Ethylene response factor c2	-1.08	3.88
Solyc03g093610	Ethylene response factor a2	-	-3.53

“-” represents no significant difference. The fold change value is represented by the log_2_ ratio.

Ethylene signal transduction plays crucial roles in mediating the biochemical changes caused by ethylene. At 1 DAT, the expressions of the most genes involved in ethylene signal transduction in auxin-treated samples showed no difference compared to the control except *SlERF*.*c2* (Solyc04g014530) and *SlERF*.*c5* (Solyc02g077370) (Fig 5, Table 2). However, at 7 DAT, expressions of *Nr* (never-ripe) (Solyc09g075440) and *ETR4* (ethylene receptor) (Solyc06g053710), which showed higher levels (RPKM > 50) than the other ethylene receptors in the samples (S2 Table), were dramatically inhibited by auxin. The transcript levels of two EIN3 binding F-Box proteins-encoding genes (EBF) (Solyc07g008250, Solyc12g009560), which mediated ethylene signal transduction via the proteolysis of the transcription factor EIN3, were down-regulated in auxin-treated samples at 7 DAT. Moreover, *Sl-ERF*.*c2* (Solyc04g014530) and *Sl-ERF*.*d1* (Solyc04g051360) were the most significantly induced and repressed member of the Ethylene-responsive transcription factors (ERF) family in AX7d, respectively.

### Expression of genes involved in indoleacetic acid (IAA) synthesis and signal transduction

**Fig 6:**
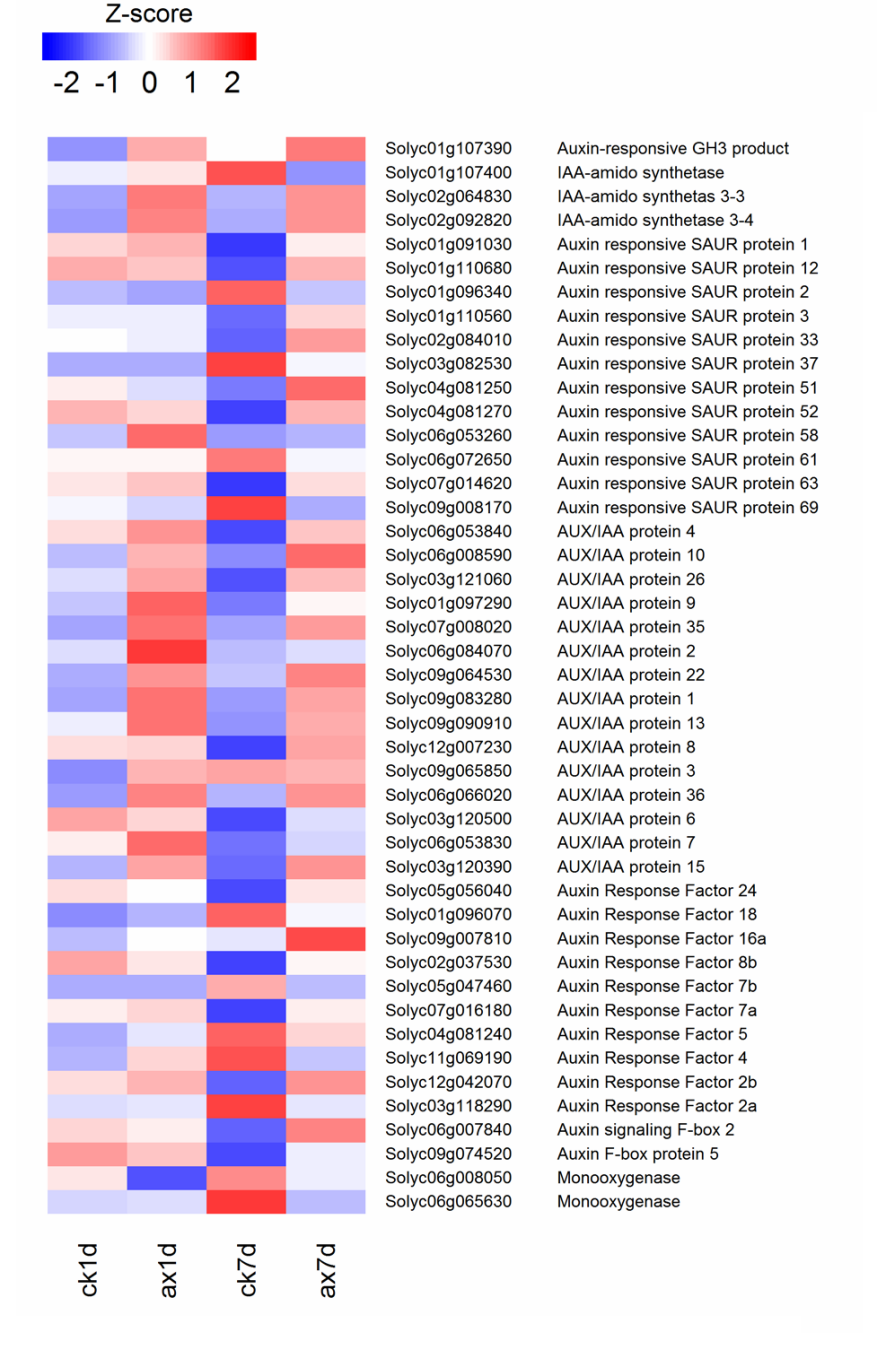
Heat map of expression levels of the DEGs involved in IAA synthesis and signal transduction. The RPKM values were normalized with log_2_ (RPKM+1) and converted to *Z*-scores to scale the expression levels of DEGs involved in ethylene synthesis and signal transduction. Red indicates high expression, while blue indicates low expression.

Forty-five DGEs related to IAA biosynthesis and signal transduction were identified (Fig 6, Table 3). In the IAA biosynthesis pathway, of the two monooxygenases, the one with the highest abundance (Solyc06g008050) (S2 Table) was down-regulated by auxin at both 1 and 7 DAT. Auxin response factors (ARF) regulate the expressions of auxin-responsive genes, which contain auxin response elements in the promoter regions. At 1 DAT, most *ARF* genes were stably expressed in the auxin group fruit, except for one (Solyc11g069190), which changed slightly. At 7 DAT, among ten differentially expressed *ARF* genes, five (Solyc12g042070, Solyc07g016180, Solyc02g037530, Solyc09g007810, Solyc05g056040) were up-regulated, and the others (Solyc03g118290, Solyc11g069190, Solyc04g081240, Solyc05g047460, Solyc01g096070) were down-regulated in auxin-treated fruit. Three early auxin response gene families, auxin/indole-3-acetic acid (Aux/IAA), Gretchen Hagen-3 (*GH3*), and small auxin-up RNA (*SAUR*), were directly regulated by ARFs. Aux/IAA proteins are negative regulators of auxin signal transduction that bind to ARFs. Fifteen *Aux/IAA* genes were significantly up-regulated by auxin and maintained high expression levels at 7 DAT. For the *GH3* gene family, four *GH3* genes (Solyc02g092820, Solyc02g064830, Solyc01g107400, Solyc01g107390) were up-regulated in AX1d *vs*. CK1d, and three of them showed the same pattern in AX7d *vs*. CK7d except one (Solyc01g107400). Moreover, the expression levels of two *GH3* genes (Solyc01g107400, Solyc01g107390) were higher in CK7d than those in CK1d. For the *SAUR* gene family, no differences were observed in most *SAUR* genes in AX1d *vs*. CK1d except *SlSAUR58* (Solyc06g053260).

**Table 3:** DEGs involved in IAA synthesis and signal transduction.

		Fold change (log_2_ ratio)	
Gene ID	Annotation	AX1d *vs*. CK1d	AX1d *vs*. CK1d
Solyc06g065630	Monooxygenase	-	-5.57
Solyc06g008050	Monooxygenase	-1.45	-1.14
Solyc09g074520	Auxin F-box protein 5	-	1.22
Solyc06g007840	Auxin signaling F-box 2	-	1.58
Solyc03g118290	Auxin response factor 2a	-	-1.18
Solyc12g042070	Auxin response factor 2b	-	0.50
Solyc11g069190	Auxin response factor 4	0.59	-1.25
Solyc04g081240	Auxin response factor 5	-	-0.95
Solyc07g016180	Auxin response factor 7a	-	1.71
Solyc05g047460	Auxin response factor 7b	-	-0.79
Solyc02g037530	Auxin response factor 8b	-	0.83
Solyc09g007810	Auxin response factor 16a	-	0.57
Solyc01g096070	Auxin response factor 18	-	-1.27
Solyc05g056040	Auxin response factor 24	-	1.11
Solyc03g120390	AUX/IAA protein 15	2.34	3.26
Solyc06g053830	AUX/IAA protein 7	-	2.48
Solyc03g120500	AUX/IAA protein 6	-0.42	1.23
Solyc06g066020	AUX/IAA protein 36	8.87	4.38
Solyc09g065850	AUX/IAA protein 3	4.02	-0.47
Solyc12g007230	AUX/IAA protein 8	-	1.71
Solyc09g090910	AUX/IAA protein 13	3.26	3.60
Solyc09g083280	AUX/IAA protein 1	3.67	3.14
Solyc09g064530	AUX/IAA protein 22	4.11	3.52
Solyc06g084070	AUX/IAA protein 2	3.74	-
Solyc07g008020	AUX/IAA protein 35	6.79	5.94
Solyc01g097290	AUX/IAA protein 9	3.97	4.17
Solyc03g121060	AUX/IAA protein 26	-	2.67
Solyc06g008590	AUX/IAA protein 10	2.45	7.20
Solyc06g053840	AUX/IAA protein 4	0.88	2.59
Solyc09g008170	SAUR protein 69	-	-9.54
Solyc07g014620	SAUR protein 63	-	2.89
Solyc06g072650	SAUR protein 61	-	-1.71
Solyc06g053260	SAUR protein 58	3.32	-
Solyc04g081270	SAUR protein 52	-	3.57
Solyc04g081250	SAUR protein 51	-	2.42
Solyc03g082530	SAUR protein 37	-	-4.44
Solyc02g084010	SAUR protein 33	-	2.41
Solyc01g110560	SAUR protein 3	-	2.67
Solyc01g096340	SAUR protein 2	-	-1.63
Solyc01g110680	SAUR protein 12	-	2.17
Solyc01g091030	SAUR protein 1	-	4.23
Solyc02g092820	IAA-amido synthetase 3–4	13.60	9.81
Solyc02g064830	IAA-amido synthetase 3–3	6.75	4.51
Solyc01g107400	IAA-amido synthetase	1.04	-5.50
Solyc01g107390	Auxin-responsive GH3 product	3.52	1.98

### Expression of genes involved in carotenoid metabolism

The color transition from green to red, with the accumulation of carotenoid and lycopene, is a major change during ripening of tomato fruit. We observed a significant inhibition of color change in auxin-treated fruit (Fig 1E). Therefore, we investigated the expressions of the genes encoding the enzymes associated with carotenoid metabolism. Most genes upstream of lycopene were down-regulated, with log_2_ fold changes ranging from -3.35 to -0.98 in AX7d compared with CK7d, except *SlPSY2* (phytoene synthase 2) (Solyc02g081330) (Table 4). In the downstream carotenoid metabolic pathway, β-carotene is generated from lycopene and finally converted to neoxanthin by various enzymes. Two genes encoding lycopene beta-cyclase (Solyc10g079480, Solyc04g040190) and one gene encoding zeaxanthin epoxidase (*SlZEP*, Solyc10g083790) were up-regulated whereas the genes encoding β-carotene hydroxylase (Solyc03g007960) and carotenoid isomerase (Solyc10g081650) were down-regulated in AX7d *vs*. CK7d.

**Table 4:** DEGs associated with carotenoid metabolism.

		Fold change (log_2_ ratio)	
Gene ID	Annotation	AX1d *vs*. CK1d	AX1d *vs*. CK1d
Solyc02g090890	Zeaxanthin epoxidase	-	2.65
Solyc01g097810	Zeta-carotene desaturase	-	-1.00
Solyc02g081330	Phytoene synthase 2	-	1.46
Solyc03g031860	Phytoene synthase 1	-	-3.36
Solyc10g079480	Beta-lycopene cyclase	-	2.27
Solyc04g040190	Lycopene beta-cyclase	-	1.06
Solyc06g074240	Lycopene beta cyclase	-	0.00
Solyc10g083790	Cytochrome P450	-	0.69
Solyc10g081650	Carotenoid isomerase	-	-0.98
Solyc03g007960	Beta-carotene hydroxylase-2	-	-3.77

### Expression of genes involved in cell wall degradation

In the present work, fruit softening was observed to be delayed by auxin treatment (Fig 1B). Polygalacturonase (PG), pectinesterase (PME), β-xylosidase, pectate lyase and expansin (EXP) play crucial roles in cell wall degradation. At 1 DAT, one (Solyc10g080210) of the genes encoding a polygalacturonase precursor was found to be repressed by exogenous auxin. At 7 DAT, most of the genes encoding these enzymes were significantly down-regulated in AX7d *vs*. CK7d (Table 5). In contrast to the other members of the expansin-coding genes, *SlEXP1* (Solyc06g051800) was greatly reduced in AX7d *vs*. CK7d.

**Table 5:** DEGs associated with cell wall degradation.

		Fold change (log_2_ ratio)	
Gene ID	Annotation	AX1d *vs*. CK1d	AX1d *vs*. CK1d
Solyc10g080210	Polygalacturonase-2 precursor	-1.67	-10.17
Solyc06g060170	Probable polygalacturonase-like	-	-1.88
Solyc05g005170	Polygalacturonase	-	-8.58
Solyc12g098340	Probable pectinesterase 29-like	-	0.65
Solyc03g083360	Probable pectinesterase	-	-1.17
Solyc03g078090	Probable pectinesterase	-	-6.10
Solyc07g017600	Pectinesterase	-	-2.31
Solyc08g081620	Endo-1,4-beta-glucanase precursor	-	-2.63
Solyc09g010210	Endo-1,4-beta-glucanase precursor	-	-2.77
Solyc02g091680	Probable beta-D-xylosidase 6-like	-	-1.12
Solyc01g104950	Beta-xylosidase	-	-2.29
Solyc10g047030	Beta-D-xylosidase 1 precursor	-	-6.44
Solyc09g005850	Probable pectate lyase 4-like	-	-1.22
Solyc03g111690	Probable pectate lyase 18-like	-	-4.67
Solyc09g091430	Probable pectate lyase 15-like	-	-6.87
Solyc03g031840	Expansin precursor	-	1.58
Solyc06g051800	Expansin 1	-	-1.28
Solyc10g086520	Expansin precursor 6	-	2.31
Solyc02g088100	Expansin precursor 5	-	1.87

### Expression of genes involved in energy metabolism

**Fig 7:**
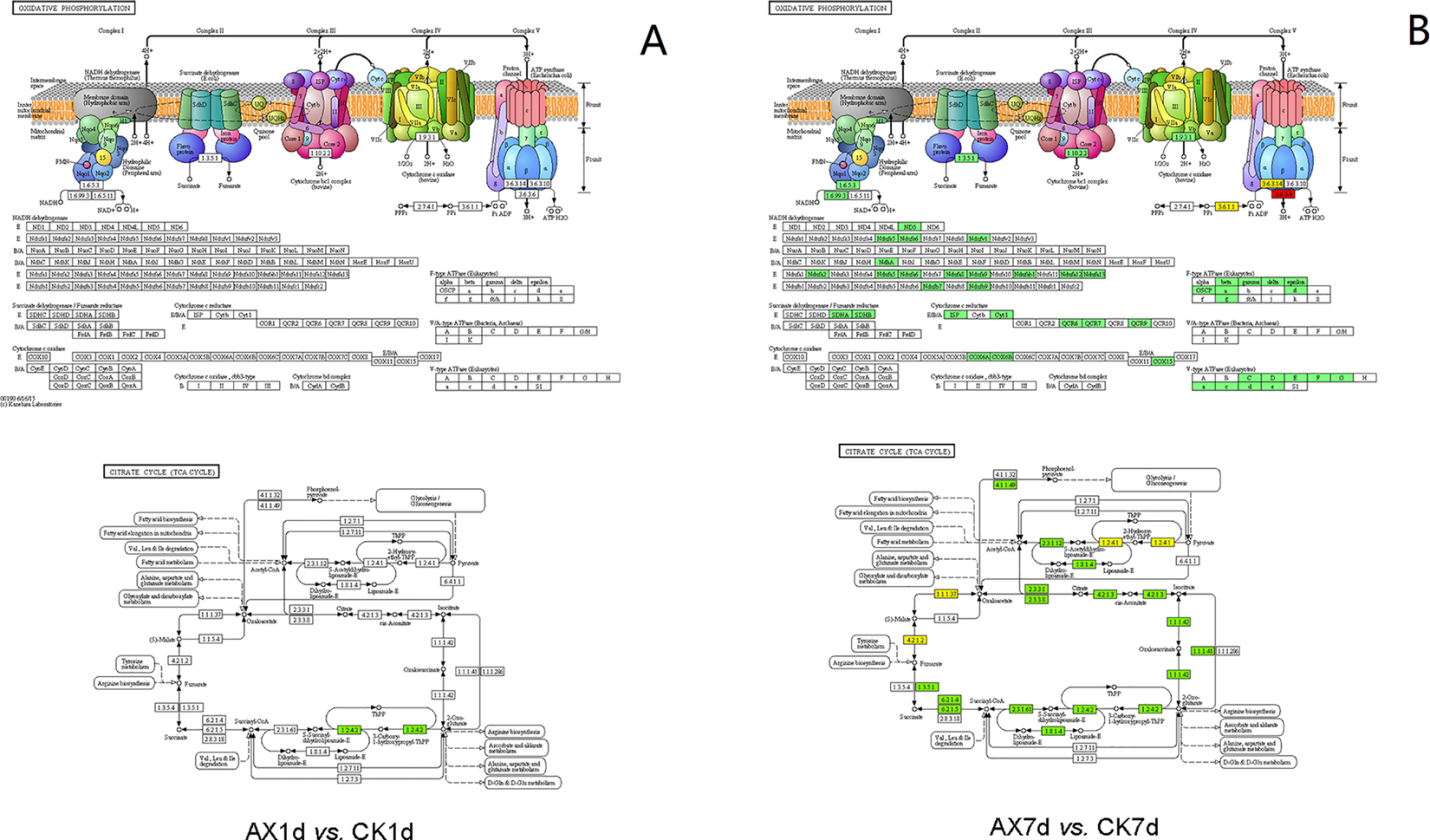
The expression of DEGs in oxidative phosphorylation and the citrate cycle. The expression patterns of DEGs involved in oxidative phosphorylation and the citrate cycle in the comparison groups (A) AX1d *vs*. CK1d and (B) AX7d *vs*. CK7d. Red and green boxes represent the genes that are up-regulated and down-regulated, respectively. The yellow box represents the genes that are both up- and down-regulated.

Fruit ripening is a highly complex process that requires a sufficient energy supply for the synthesis of a large number of mRNAs, proteins, flavor compounds, and other molecules. In the comparison group AX1d *vs*. CK1d, only one gene (Solyc04g011350) associated with the citrate cycle (TCA cycle) was slightly depressed, and no DEGs were enriched in the oxidative phosphorylation pathway (Fig 7A). However, most of the genes involved in the TCA cycle and oxidative phosphorylation pathway were significantly down-regulated in AX7d *vs*. CK7d (Fig 7B). These results indicate that auxin may inhibit fruit respiration rate and reduce fruit vitality during ripening.

### Validation of RNA-Seq data by RT-qPCR

The expression patterns of twelve genes that were randomly selected from the RNA-Seq data were validated by RT-qPCR. The results of linear regression analysis indicated a high correlation (*r*^*2*^ = 0.94) between the data of RT-qPCR and RNA-Seq (S1 Fig).

## Discussion

### The short-term and long-term effects of exogenous auxin on tomato fruit

Auxin regulates many plant physiological processes by modulating the expressions of auxin-response genes. Three gene families, *AUX/IAA*, *GH3* and *SAUR*, exhibit rapid changes in expression in response to exogenous auxin, indicating the necessity for a quick response to auxin signaling in the plant [37]. However, many auxin-induced physiological or molecular changes did not appear in a short-term period, suggesting the importance of the long-term effects of auxin [8–10]. Here, we analyzed the short-term (1 DAT) and long-term (7 DAT) effects of auxin on the biology and molecular processes in postharvest tomato fruit.

For short-term effects, we found that glutathione metabolism was the most enriched KEGG pathway in AX1d *vs*. CK1d (Fig 4). Thirteen genes encoding glutathione S-transferases (GSTs) were strongly up-regulated by auxin treatment. GSTs catalyze the reaction converting glutathione (GSH) to R-S-glutathione and are essential enzymes in plant stress resistance that protect the tissues against oxidative stress, toxins, herbicide stress, and other stressors [38–40]. The improvement of stress tolerance and resistance by overexpression of GST-encoding genes has been reported in other studies [41–43]. Additionally, the expression of GSTs can also be induced by auxin [44, 45] and many other phytohormones [46, 47], indicating the involvement of GSTs in the hormone signal response. In addition to GSTs, twelve and four members of the *AUX/IAA* and *GH3* gene family were up-regulated with various log_2_ fold changes in auxin-treated fruit, respectively (Table 3). Most of these auxin response genes decreased at 7 DAT (Table 3), suggesting that they may mediate the short-time effects of auxin. With regard to TFs, *NAC* (*NAM*, *ATAF*, and *CUC*) family genes were the major regulated TF genes in AX1d *vs*. CK1d (S2 Fig, S9 Table). One *NAC* family member, *SlNAM3* (Solyc06g069710), was the most highly induced TF gene in auxin-treated fruit (S9 Table). NAC TFs participate in various biological processes, including stress resistance [48]. Overexpression of NAC TFs can enhance plant drought resistance and salt tolerance [49–51]. Moreover, high levels of auxin can also result in stress in the plant, which involves inducing defense- and detoxication-related genes to maintain auxin homeostasis. Based on these reports, we hypothesize that the improvement of defense and stress resistance functions may be one of the major short-term effects of auxin in postharvest tomato fruit.

For the long-term effects, we observed that most of the genes associated with carotenoid metabolism, cell wall degradation and energy metabolism were down-regulated in auxin-treated fruit. In tomato, *SlPSY2* (Solyc02g081330), which has been reported to be responsible for carotenoid accumulation in root and green tissues [52], was up-regulated by auxin. In contrast, *SlPSY1* (Solyc03g031860) is functional during fruit ripening, and its level are higher than that of *SlPSY2* in fruit [52]. The expression of *SlPSY1* was strongly repressed by auxin at 7 DAT, suggesting that auxin has a longer inhibitory effect than that described in the previous report [9]. Regarding the effects on fruit softening, auxin may play contradictory roles in different fruits and development stages. Auxin appears to promote softening of peach [53] and apple [54, 55], but it maintains the firmness of strawberry [56] and citrus [57]. In the present study, we confirmed that applying auxin to tomato fruit before ripening repressed the genes associated with cell wall degradation. In addition, another significant long-term effect of auxin was the repression of fruit energy metabolism. The auxin-mediated restriction on respiration has been observed in apple [58] and loquat fruitlets [59], but reports examining the direct effects of auxin on energy metabolism are still lacking. Based on our transcriptome sequencing results, the inhibition of energy metabolism by auxin was also a long-term effect. The expressions of genes associated with the TCA cycle and oxidative phosphorylation in auxin-treated fruit showed a marked decrease at 7 DAT but almost no difference at 1 DAT compared with the control fruit (Fig 7). Notably, compared with the fruit at AX1d, most of oxidative phosphorylation-related genes were down-regulated at AX7d, suggesting a homeostatic regulation of the energy supply corresponding to the lower energy demand due to delayed ripening.

### Auxin may maintain system 1 ethylene synthesis and prevent the initiation of system 2 ethylene

The transition from system 1 to system 2 ethylene production is essential for climacteric fruit ripening. In tomato, at least nine *ACS* genes (*SlACS1a*, *SlACS1b*, *SlACS2-8*) and five *ACO* genes (*SLACO1-5*) have been identified, and their expression patterns have been studied [60–64]. *SlACS1a* and *SlACS6* are responsible for system 1 ethylene production [60, 65]. The transcript levels of these two genes declined in mature green tomato fruit after treatment with exogenous ethylene, demonstrating the negative effect of ethylene on the regulation of these two genes [65]. Moreover, *SlACS1a* also plays an important role in the transition from system 1 to system 2 ethylene [65]. Two other members of the *ACS* gene family, *SlACS2* and *SlACS4*, which are repressed in the pre-climacteric period but greatly induced at the ripening stage, are responsible for system 2 ethylene synthesis [60, 65, 66]. In addition, *SlACO1*, *SlACO3* and *SlACO4* are expressed at low levels before the climacteric period but are increased at the onset of the ripening stage [67]. In the present study, we found that the system 2 ethylene initiated at 7 DAT and the expressions of *SlACS1a* (Solyc08g081550), *SlACS1b* (Solyc08g081540), *SlACS2* (Solyc01g095080), *SlACS*4 (Solyc05g050010), *SlACO1* (Solyc07g049530), *SlACO3* (Solyc09g089580), *ACO4* (Solyc07g049550) and *SlACO5* (Solyc07g026650) were increased. However, in auxin-treated fruit, the expressions of both *ACS* and *ACO*, which are associated with system 2 ethylene synthesis, were still at low levels (Table 2), resulting in low ethylene production (Fig 1E). These results suggest that auxin application in the pre-climacteric period may delay the onset of system 2 ethylene biosynthesis. We also observed that *SlACS6* (Solyc08g008100), a crucial *ACS* gene related to system 1 ethylene synthesis, was greatly induced in auxin-treated fruit at 7 DAT (Table 2, Fig 5), indicating that auxin may maintain system 1 ethylene synthesis.

Previous studies have shown that epigenetic remodeling plays a crucial role in the transition from ethylene system 1 to ethylene system 2 [68, 69]. The methylation level of promoters affects the binding affinity of transcription factors, leading to the modulation of gene expression. The promoter region of the *ACS* and *ACO* genes is hypermethylated at the developing stage and demethylated during ripening [69]. In addition, the expressions of many DNA methyltransferase-coding genes are also modulated by 1-methylcyclopropene (1-MCP), an ethylene inhibitor, demonstrating the complex interaction between ethylene and methylation [68]. In control fruit, from 1 DAT to 7 DAT, we observed a significant increase in three DNA demethylase-coding genes (Solyc09g009080, Solyc10g083630, Solyc11g007580), along with a decrease in five cytosine-5 DNA methyltransferase-coding genes (Solyc11g030600, Solyc04g005250, Solyc01g006100, Solyc12g100330, Solyc02g062740) (Table 6). Compared with the control, most of the cytosine-5 DNA methyltransferase-encoding genes and DNA demethylase-encoding genes were induced and repressed in auxin-treated fruit, respectively (Table 6). This may contribute to the maintenance of high methylation levels in the nucleus, inhibiting the demethylation of system 2 ethylene-related genes and delaying the onset of climacteric ethylene.

**Table 6:** DEGs associated with DNA methylation.

		Fold change (log_2_ ratio)		
Gene ID	Annotation	AX1d *vs*. CK1d	AX1d *vs*. CK1d	AX1d *vs*. CK1d
Solyc09g009080	DNA demethylase1	-	-	0.87
Solyc10g083630	DNA demethylase 2	-	-1.43	1.01
Solyc11g007580	DNA demethylase 3	-0.49	-1.84	1.76
Solyc11g030600	Cytosine-5 DNA methyltransferase	-	0.89	-1.36
Solyc04g005250	Cytosine-5 DNA methyltransferase 1	-	1.25	-1.22
Solyc01g006100	Cytosine-5 DNA methyltransferase 1L	-	-	-6.36
Solyc12g100330	Cytosine-5 DNA methyltransferase 3L	-	-0.49	-0.69
Solyc02g062740	Cytosine-5 DNA methyltransferase 5	-	-	-0.43

### The crosstalk between auxin and ethylene in the fruit ripening process

The crosstalk between auxin and ethylene may occur through the expression of genes that contain both auxin response elements (AuxRE) and ethylene response motifs (ERELEE4) in their promoter or respond to both of the phytohormones [17]. Many *ARF* and *ERF* promoters contain several AuxRE and ERELEE4 motifs, allowing these transcription factors to modulate the expression of each other [20, 70]. In *Arabidopsis*, *AtARF7* and *AtARF19* have been reported to be regulators involved in the ethylene response [71]. *CpARF7*, a homolog of *AtARF7* in papaya (*Carica papaya L*.), was also shown to be involved in fruit ripening via the regulation of ethylene signaling [72]. In addition to the regulation by ethylene, several *ERF* genes are also induced by auxin [7, 21, 73]. The existence of ethylene-auxin interactions during fruit ripening has been demonstrated in a previous study [7]. According to our results, eight *ERF* genes, especially *SlERF*.*a2* (Solyc03g093610), *SlERF*.*d1* (Solyc04g051360), *SlERF*.*d3* (Solyc01g108240) and *SlERF*.*d4* (Solyc10g050970), were dramatically repressed by auxin (Fig 5). However, of the five auxin-induced *ERF* genes, the expression of *SlERFc2* (Solyc04g014530) and *SlERFe3* (Solyc06g082590) showed the highest increase in the AX7d *vs*. CK7d comparison. These results indicate that auxin participates in the ethylene response by modulating *ERF* gene expression. With regard to *ARF* genes, five (*SlARF2a*, Solyc03g118290; *SlARF4*, Solyc11g069190; *SlARF5*, Solyc04g081240; *SlARF7b*, Solyc05g047460; *SlARF18*, Solyc01g096070) were up-regulated and three (*SlARF7a*, Solyc07g016180; *SlARF8b*, Solyc02g037530; *SlARF24*, Solyc05g056040) were down-regulated during ripening (Fig 6). These *ARF* genes may play contrary roles in ripening. For example, *SlARF4* plays an important role in the control of sugar and chlorophyll metabolism. Down-regulation of *SlARF4* results in dark green fruit and blocks ripening [23, 24]. The ARF genes *SlARF2a* and *SlARF2b* are involved in the regulation of many key *TF* genes, such as *RIN*, *CNR* and *NOR*, and are down-regulated, resulting in the inhibition of ripening [74]. Most of the *ARF* gene expression patterns were consistent with a previous study [20], except *SlARF4*. This slight difference may be due to the tomato cultivar. In addition to the ARF and ERF genes, the *TF* genes associated with ripening were differentially regulated by auxin and ethylene. Three crucial ripening-related *TF* genes, *RIN* (Solyc05g012020), *CNR* (Solyc02g077920) and *TAGL1* (Solyc07g055920), were all repressed by auxin (*RIN*, log_2_ ratio: -2.89; *CNR*, log_2_ ratio: -2.16; *TAGL1*, log_2_ ratio: -0.64) (S4 Table). These findings suggest that auxin may regulate the expression of ripening-related *TF* genes, leading to changes in carotenoid biosynthesis, cell wall degradation and energy metabolism, finally delaying the ripening process.

**Fig 8:**
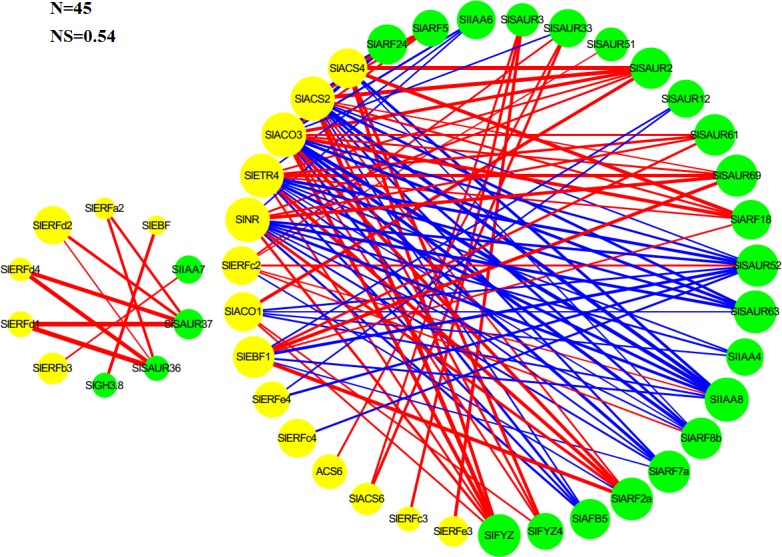
Correlation network of auxin- and ethylene-related genes. “NS” and “N” indicate network strength and the number of nodes, respectively. Node strength and network strength were calculated as previously described [35] and are listed in S10 Table. Each node represents an auxin-related gene (green) or an ethylene-related gene (yellow). Node size is proportional to node strength. Red and blue lines represent the positive and negative correlation between two genes, respectively. Line thickness is proportional to the absolute value of the correlation coefficient (*r*). Only high-level correlations (∣*r*∣< 0.75) between auxin- and ethylene-related genes are shown in the network.

We also performed correlation network analysis to assess the potential interactions between auxin and ethylene as previously described [35]. Only correlations between auxin- and ethylene-related genes are shown in this network (Fig 8). Five auxin-related nodes with high correlation values (node strength > 0.6) (S10 Table), *SlSAUR63*, *SlSAUR52*, *SlIAA8*, *SlARF7a*, and *SlARF8b*, predominantly showed negative correlations with ethylene-related genes, whereas *SlSAUR69*, *SlSAUR2* and *SlARF2a* exhibited positive correlations. Although the auxin response factor gene *SlARF2a* has been shown to modulate tomato ripening [74], the functions of the other genes showing strong correlations to ethylene-related genes should be investigated further.

**Fig 9:**
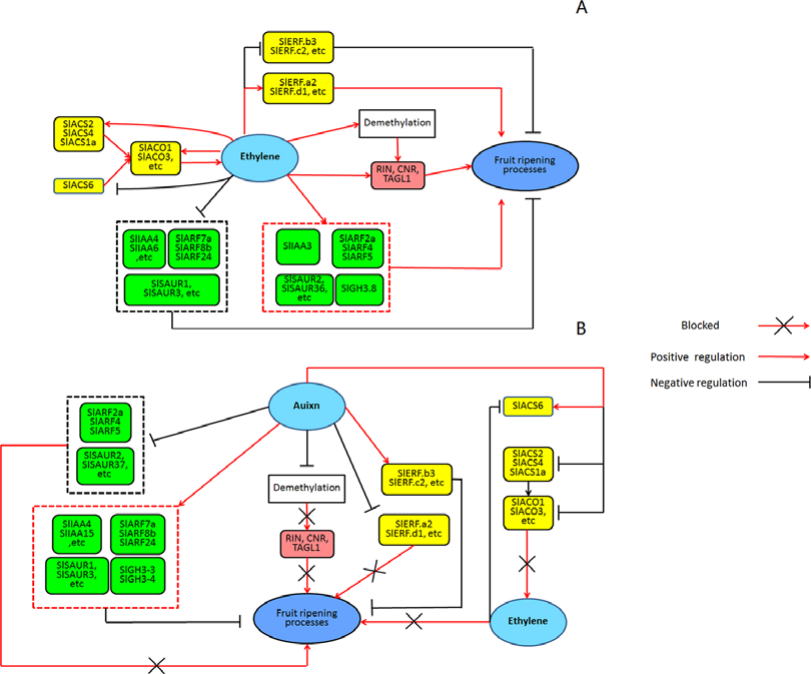
Model for the regulation of exogenous auxin on fruit ripening cooperating with ethylene. (A) Normal ripening process with the regulation of ethylene and ripening-related TFs (RIN, CNR, TAGL1, and others). (B) Exogenous auxin inhibits ripening by modulating epigenomic remodeling, ethylene synthesis and the expression of auxin response genes.

Based on our study, we established a model to explore the effects of ethylene-auxin crosstalk on fruit ripening (Fig 9). During normal ripening, the methylation level in the nucleus is deceased, leading to the activation of TFs (RIN, CNR, TAGL1) and the transition to system 2 ethylene. The genes involved in ethylene (*SlERF*.*a2*, *SlERF*.*b3*, and others) or auxin (*SlIAA3*, *SlARF7a*, and others) signal transduction are differentially expressed to modulate the ripening process (Fig 9A). After treatment of exogenous auxin, demethylation is inhibited, leading to a block of the function of the TFs (RIN, CNR, TAGL1). The synthesis of system 2 ethylene (auto stimulatory) is inhibited, whereas system 1 ethylene (auto inhibitory) is maintained. Meanwhile, the expression of the auxin- or ethylene-related genes that are up- or down-regulated in normal ripening are disturbed by exogenous auxin, leading to a final delay in ripening (Fig 9B).

## Conclusions

Auxin has been shown to delay the fruit ripening process. Our present work revealed possible mechanisms for the modulation of ripening by auxin-ethylene interactions. Auxin treatment up-regulated the expression of auxin response genes, GST-encoding genes and stress tolerance-related *TF* genes and induced the recruitment of a large number of defense-associated genes to improve stress resistance and maintain auxin homeostasis. During the subsequent period, auxin enhanced the transcript levels of the genes involved in system 1 ethylene synthesis and maintained the high methylation level in the nucleus to repress the expression of system 2 ethylene synthesis-related genes. Finally, the expressions of the genes associated with carotenoid metabolism, cell wall degradation and energy metabolism were strongly repressed, and the ripening process was retarded. Additionally, the expression patterns of the genes involved in ethylene biosynthesis and signal transduction were markedly disturbed by exogenous auxin. The results of correlation network analysis revealed a strong correlation between auxin- and ethylene-related genes, suggesting significant crosstalk between auxin and ethylene during tomato ripening.

## Supporting Information

S1 Fig Correlation analysis of gene expression data from RNA-Seq and RT-qPCR. (TIF)

S2 Fig Differentially expressed TF genes. Differentially expressed TF genes in response to exogenous auxin at (A) 1 DAT and (B) 7 DAT.
(TIF)

S1 Table Primers used in the RT-qPCR assays. (XLSX)

S2 Table The expression levels (RPKM) of genes in all samples. (XLSX)

S3 Table DEGs in response to exogenous auxin at 1 DAT. (XLSX)

S4 Table DEGs in response to exogenous auxin at 7 DAT. (XLSX)

S5 Table GO enrichment analysis of DEGs in the comparison group AX1d *vs*. CK1d. (XLSX)

S6 Table GO enrichment analysis of DEGs in the comparison group AX7d *vs*. CK7d. (XLSX)

S7 Table KEGG pathway enrichment analysis of DEGs in the comparison group AX1d *vs*. CK1d. (XLSX)

S8 Table KEGG pathway enrichment analysis of DEGs in the comparison group AX7d *vs*. CK7d. (XLSX)

S9 Table Top ten differentially expressed TF genes in response to exogenous auxin. (XLSX)

S10 Table Node strengths of the correlation networks. (XLSX)
