# Supplementary figures and images for: Comprehensive RNA-Seq Analysis on the Regulation of Tomato Ripening by Exogenous Auxin
Source: PLoS One. 2016 May 26;11(5):e0156453. doi: 10.1371/journal.pone.0156453 (PMC4881990; doi:10.1371/journal.pone.0156453)

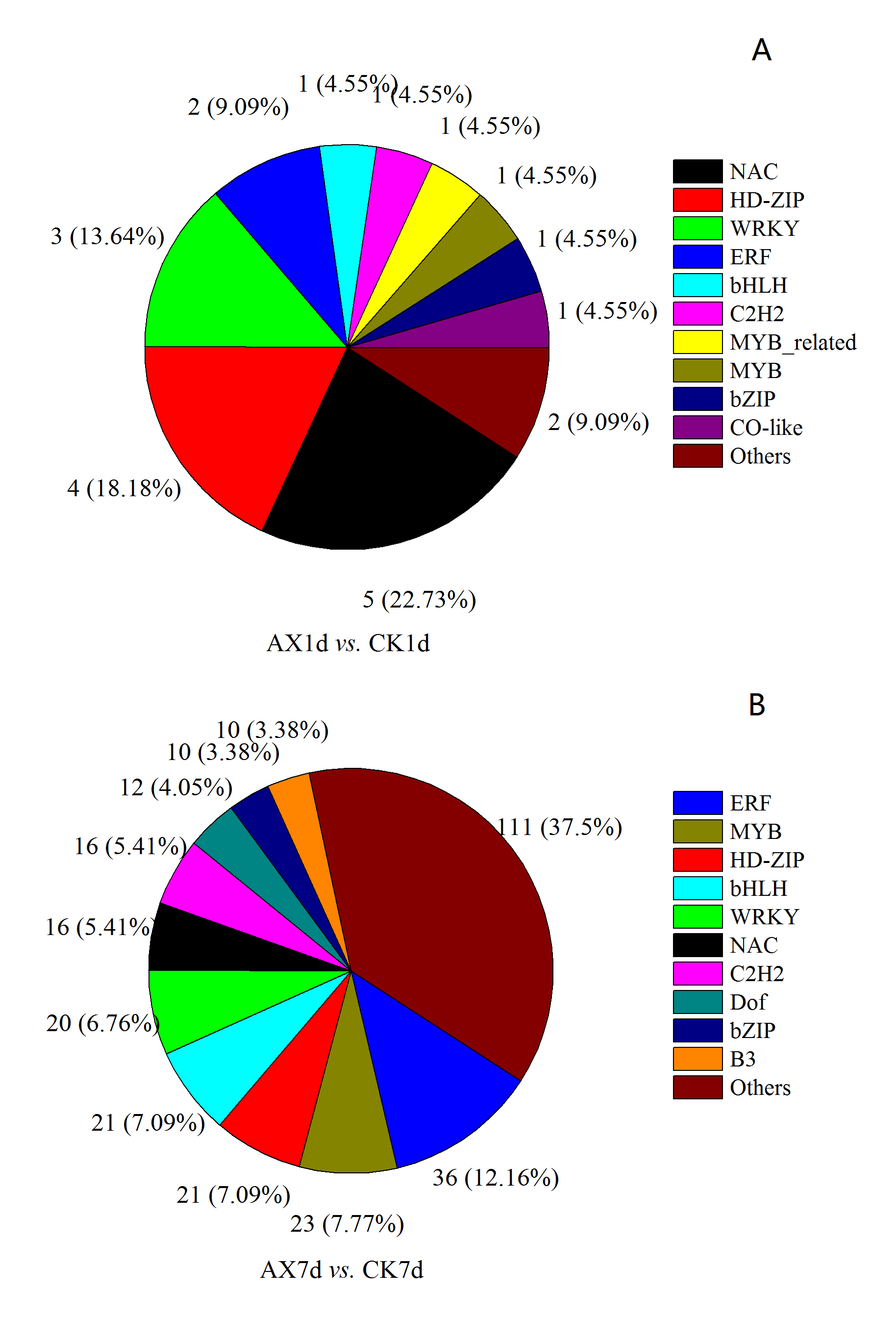

Supplement: S2 Fig — Differentially expressed TF genes in response to exogenous auxin at (A) 1 DAT and (B) 7 DAT. (TIF) [file pone.0156453.s002.tif]
